# Flexor Carpi Radialis Muscle: Anatomic Features and Electromyography Technique Under Ultrasound Control

**DOI:** 10.7759/cureus.27936

**Published:** 2022-08-12

**Authors:** Fatma Elleuch, Wafa Elleuch, Ahmed Mohameden, Houcem Harbi, Sameh Ghroubi, Habib Elleuch

**Affiliations:** 1 Department of Physiology, Habib Bourguiba Hospital, University of Sfax, Sfax, TUN; 2 Department of Physical Medicine and Rehabilitation, Habib Bourguiba Hospital, University of Sfax, Sfax, TUN; 3 Department of General Surgery, Research Laboratory LR21ES04, Habib Bourguiba Hospital, University of Sfax, Sfax, TUN; 4 Department of Physical Medicine and Rehabilitation, Research Laboratory LR20ES09, Habib Bourguiba Hospital, University of Sfax, Sfax, TUN

**Keywords:** extensors' deficiency, electromyography, ultrasound, anatomy, flexor radialis carpi muscle

## Abstract

The electromyographic assessment of the flexor carpi radialis muscle (FCRM) in the context of an upper limb extensors’ deficiency helps physicians in differentiating radial nerve damage from C7 radicular impairment. Ultrasound (US)-guided electromyography (EMG) is mandatory to locate this muscle, particularly in the case of muscle atrophy, denervation, and neuromuscular disorders. The aim of this manuscript is to illustrate the anatomical specific features of the FCRM and the technical procedure of FCRM EMG with US guidance.

## Introduction

Nowadays, practitioners frequently resort to ultrasound (US) guidance to perform electromyography (EMG) because this significantly increases its accuracy and, therefore, diagnostic performance. Thus, teaching US guidance within EMG education would improve patient care and resident training.

The use of US requires expertise, particularly for deep, thin or pathological muscles that are considered key in diagnosing the neural damage level [1, 2]. For example, the flexor carpi radialis muscle (FCRM) is considered a key muscle in diagnosing the neurological lesion causing upper limb extensors’ deficiency [3].

The aim of this manuscript is to illustrate the anatomical characteristics of the FCRM and the EMG technique of this muscle under ultrasound control.

## Technical report

A practitioner requires a good knowledge of the US anatomy of the forearm so that he can locate the FCRM and proceed to its evaluation with needle EMG.

**Figure 1:**
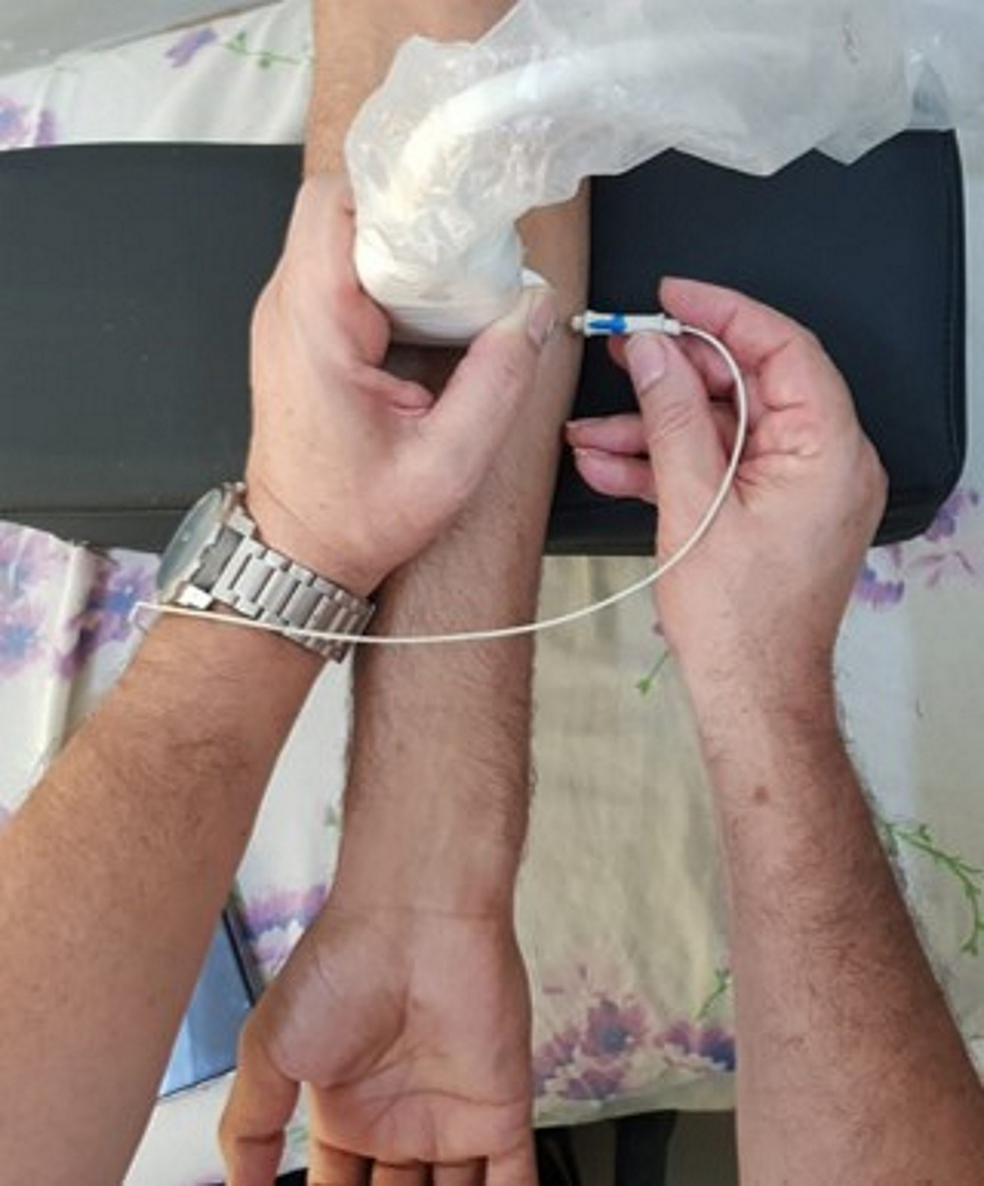
EMG needle insertion under US control. The US probe is placed at the junction of the upper and middle thirds of the anterior side of the forearm. The EMG needle is inserted at the medial edge of the forearm and 2 cm away from the US probe. This needle should then follow a sloped path to the FCRM in the plane of the US section so as to be entirely viewed: this is called the US in-plane technique. US: Ultrasound; FCRM: Flexor carpi radialis muscle; EMG: Electromyography. Note: In this figure, a medical resident has voluntarily given his consent for demonstration purposes.

**Figure 2:**
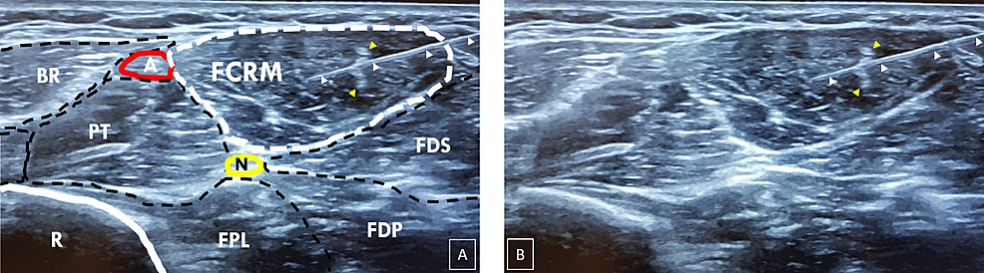
US section at the junction of the upper and middle thirds of the anterior side of the forearm with (panel B) and without delineation of muscles (panel A). The FCRM is located in the middle of the superficial part of the anterior compartment of the forearm. The EMG needle (white arrowheads) is entirely viewed and inserted in the FCRM. This muscle has a characteristic central septum (yellow arrowheads). BR: Brachioradialis muscle; FDS: Flexor digitorum superficialis muscle; FDP: Flexor digitorum profundus muscle; FPL: Flexor pollicis longus muscle; PT: Pronator teres muscle; N: Median nerve; A: Radial artery; R: Radius; FCRM: Flexor carpi radialis muscle; EMG: Electromyography. Note: These ultrasound images were taken from a voluntary medical resident who consented to the procedure.

From a technical point of view, the physician must first place the US probe at the level of the upper third of the anterior side of the forearm, as shown in Figure 1. In addition, the EMG needle should be inserted at the level of the medial side of the forearm just next to the US probe so it is completely visualized in the plane of the US section (Figure 1). The FCRM is then easily located as a central and superficial muscle of the anterior compartment of the forearm (Figure 2). The EMG needle should then be simply directed to and inserted into this muscle under US guidance (Figure 2).

## Discussion

Anatomic-specific features of the FCRM

The FCRM is a superficial muscle of the anterior forearm compartment. It originates at the level of the medial epicondyle (epitrochlea) of the humerus below the pronator teres origin. Then, it follows an inferior and lateral path. At the distal third of the forearm level, the FCRM tendon constitutes the medial limit of the radial pulse groove (laterally limited by the brachioradialis muscle). This tendon passes under the flexor retinaculum (anterior annular carpal ligament), surrounded by a serous sheath. It then passes into the lateral part of the carpal tunnel to end on the ventral side of the second and third metacarpal bases with an expansion to the trapezius bone. The FCRM is innervated by median nerve branches from the C6 to C7 roots [4]. The FCRM ensures the flexion and radial tilt of the wrist [4].

Ultrasound contribution to perform EMG

It is currently accepted that US guidance is a complementary tool for EMG [5, 6]. Several studies have also shown that US is essential for performing EMG of small and deep muscles [2, 7, 8]. For example, the study by Jin TG et al. showed that the accuracy of EMG needle placement for the extensor indicis proprius and the supinator muscle was very low without US control. The authors also stated that there is a 75% risk of anatomic mistargeting of such muscles [6]. In addition, the supinator muscle is frequently used for nerve transfer [9]. Hence, the assessment of the supinator or the extensor indicis proprius allows for differentiation between proximal radial nerve damage and posterior interosseous nerve impairment. This makes a direct impact on clinical and therapeutic management. Thus, 100% accuracy of EMG needle placement is mandatory in such cases; hence, the indisputable contribution of US feedback.

EMG detection of certain superficial and large muscles such as FCRM generally seems easy. However, this muscle can be easily confused with the pronator teres muscle adjacent to it. Occasionally, some patients cannot perform isolated contraction of the FCRM. Therefore, it would be difficult to have an EMG assessment of this muscle. In addition, in a pathological situation such as denervation (or plegic muscle), muscle atrophy, or spasticity of the extremities (poststroke situation), it would not be easy to identify the FCR; hence, ultrasound guidance makes a crucial contribution [10, 11].

Diagnostic value of FCRM

Nerve damage to the extensor muscles of the upper limb may be due to either radial nerve damage or C7 radicular impairment. Since the FCRM is the only muscle of the anterior compartment to be innervated by branches of the median nerve originating from the C7 root and not by the radial nerve, its impairment means C7 radicular damage. On the other hand, if this muscle is normal, there should be a radial nerve lesion [3]. For example, C7 radiculopathy due to C6-C7 disc herniation would lead to triceps and wrist flexors deficiency, particularly in the FCRM [12, 13]. On the other hand, radial nerve proximal lesion between the brachial plexus and nerve division would cause wrist and fingers’ extension loss without any impairment of the FCRM. This is mainly caused by either surgical trauma or humeral fracture [13, 14]. Thus, physicians consider that the FCRM is one of the key muscles in diagnosing the site of nerve damage causing upper limb extensors’ deficiency [3].

## Conclusions

Based on the particular type of innervation of the FCRM, the physician should rely on its EMG exploration in determining the level of neurological lesion causing the upper limb extensors’ deficiency. US guidance is mandatory to locate this muscle, particularly in case of muscle atrophy, denervation, and neuromuscular disorders. Practitioners must therefore master the use of musculoskeletal US.
